# Effect of *Moringa oleifera* L. Leaf Powder Addition on the Phenolic Bioaccessibility and on In Vitro Starch Digestibility of Durum Wheat Fresh Pasta

**DOI:** 10.3390/foods9050628

**Published:** 2020-05-14

**Authors:** Gabriele Rocchetti, Corrado Rizzi, Gabriella Pasini, Luigi Lucini, Gianluca Giuberti, Barbara Simonato

**Affiliations:** 1 Department for Sustainable Food Process, Università Cattolica del Sacro Cuore, Via Emilia Parmense 84, 29122 Piacenza, Italy; gabriele.rocchetti@unicatt.it (G.R.); luigi.lucini@unicatt.it (L.L.); 2 Department of Biothechnology, University of Verona, Strada Le Grazie 15, 37134 Verona, Italy; corrado.rizzi@univr.it (C.R.); barbara.simonato@univr.it (B.S.); 3 Department of Agronomy, Food, Natural Resources, Animals and Environment (DAFNAE), University of Padova, Viale dell’Università 16, 35020 Legnaro (Padova), Italy; gabriella.pasini@unipd.it

**Keywords:** *Moringa oleifera*, phenolic bioaccessibility, starch digestion, slowly digestible starch, resistant starch

## Abstract

Fresh pasta was formulated by replacing wheat semolina with 0, 5, 10, and 15 g/100 g (*w/w*) of *Moringa oleifera* L. leaf powder (MOLP). The samples (i.e., M0, M5, M10, and M15 as a function of the substitution level) were cooked by boiling. The changes in the phenolic bioaccessibility and the in vitro starch digestibility were considered. On the cooked-to-optimum samples, by means of ultra-high-performance liquid chromatography-quadrupole time-of-flight (UHPLC-QTOF) mass spectrometry, 152 polyphenols were putatively annotated with the greatest content recorded for M15 pasta, being 2.19 mg/g dry matter (*p* < 0.05). Multivariate statistics showed that stigmastanol ferulate (VIP score = 1.22) followed by isomeric forms of kaempferol (VIP scores = 1.19) and other phenolic acids (i.e., schottenol/sitosterol ferulate and 24-methylcholestanol ferulate) were the most affected compounds through the in vitro static digestion process. The inclusion of different levels of MOLP in the recipe increased the slowly digestible starch fractions and decreased the rapidly digestible starch fractions and the starch hydrolysis index of the cooked-to-optimum samples. The present results showed that MOLP could be considered a promising ingredient in fresh pasta formulation.

## 1. Introduction

Nowadays, one of the most applied strategies to increase the nutritional properties of a certain food and provide consumers with physiological functions is the incorporation of different functional ingredients during formulation [1]. This strategy would be useful to extend health benefits to the maximum number of consumers, contributing to the reduction of nutrient deficiencies, without impairing the eating habits of the population [2]. In this context, durum wheat semolina pasta, a widely consumed product, can be an excellent staple food for the addition of different bioactive compounds [3]. Indeed, pasta formulated with different sources of dietary fiber, proteins, omega-3 fatty acids, and/or bioactive compounds has been produced [4,5]. In this framework, the use of *Moringa oleifera* L. leaf powder (MOLP) in durum wheat semolina pasta formulation could be considered a promising strategy aiming to improve the overall nutritional quality of this food product.

The *Moringa oleifera* L. plant is native to India and is cultivated worldwide for its characteristic nutritional properties and for its variety of end-uses. Every part of the *Moringa oleifera* plant contains important nutrients and phytochemicals, such as vitamins, minerals, essential amino acids, bioactive compounds, and dietary fiber [6]. The leaves of Moringa are considered a valuable source of distinctive classes of polyphenols, including flavonoids, phenolic acids, and lignans [6,7]. Polyphenols have been studied for their potential health-promoting properties, including their antioxidant capacity [8,9]. However, these benefits are not only related to the content of polyphenols in a certain food, but also to their bioaccessibility, bioavailability, and bioefficacy in humans [10,11]. Therefore, MOLP polyphenols’ bioaccessibility studies seem to be essential for a first-step investigation on the potential health benefits of this plant ingredient. However, no information is available on the changes in the phenolic profiles following in vitro digestion (i.e., bioaccessibility) for MOLP-enriched cooked fresh pasta. Besides, although the inclusion of MOLP has been reported to substantially improve the nutritional value of cereal-based foods, by increasing both the protein and dietary fiber contents, none of the studies have determined if the incorporation of MOLP could also contribute to modifying the starch digestibility, at least in vitro, in real food systems (i.e., after cooking) [6].

Considering the growing interest in MOLP in food formulation [6], due to its nutrient composition and the bioactive compound profile [7,12], in this work we produced durum wheat semolina fresh pasta with different substitution levels of MOLP, being 0, 5, 10, and 15 g/100 g (*w/w*), respectively. The MOLP substitution level up to 15 g/100 g (*w/w*) was selected considering that greater levels of MOLP in the recipe could impair the food sensory as well as the technological properties [6].

To better explore the nutritional role of MOLP in fresh pasta production, the present study aimed to evaluate the effect of increasing levels of MOLP in durum wheat semolina fresh pasta by focusing on (i) the phenolic bioaccessibility and (ii) the in vitro starch digestion of cooked pasta.

## 2. Materials and Methods

### 2.1. Materials and Fresh Pasta Sample Preparation

Durum wheat semolina and dried MOLP were acquired in a local market. As reported on the label, durum wheat semolina’s nutritional composition was as follows (g/100 g product): total starch: 70.8 g; total protein: 11.0 g; total fat: 1.8 g; total dietary fiber: 3.0 g. For the dried MOLP (g/100 g product): total starch: 15.1 g; total sugars: 3.1 g; total protein: 29.9 g; total fat: 8.2 g; total dietary fiber: 30.7 g. The MOLP and durum wheat semolina had a particle size smaller than 0.2 mm.

Fresh durum wheat semolina pasta samples with 100% durum wheat semolina (control: M0) and by replacing semolina with 5, 10, and 15 g/100 g MOLP (*w/w*), obtaining the M5, M10, and M15 pasta samples were produced, respectively. The dough was made with the addition of 35% *v/w* of tap water (37 °C) to the pure semolina or the blend semolina–MOLP by using a pasta machine (Mod. Lillodue, Bottene, Italy). The mixing time was 15 min. The resulting dough was extruded through a bronze die for a spaghetti shape (0.22 cm diameter, approximately 25.0 cm length). For each recipe, three pasta production batches were produced on the same day.

### 2.2. Moisture Content, Water Activity and Pasta Cooking Properties

The moisture content of the fresh pasta samples was measured with the method 44-15A [13]. Water activity (a_w_) was measured using a Hygropalm HC2-AW-meter (Rotronic Italia, Milano) at 23 °C. The AOAC approved method 66-50 was applied for the optimum cooking time (OCT) determination [13]. In particular, samples were cooked in distilled boiling water (ratio of 1:10, *w/v*). At 30 seconds intervals, spaghetti strands were picked from the boiling water and squeezed between 2 glass slides. The OCT for each pasta sample, by definition, is the time for disappearing the white central core of the spaghetti after being squeezed between 2 glass plates.

### 2.3. Cooking Process and Experimental Details

Prior to in vitro investigations, the spaghetti (5.0 g) were cooked in boiling water (1:10 *w/v*) according to the individual OCT, drained up for 1 min, chopped with a manual meat mincer to simulate mastication, and analyzed “as eaten”. Three separate in vitro evaluations were conducted, as detailed below.

#### 2.3.1. In Vitro Static Digestion of Cooked Samples for the Evaluation of the Fate of Polyphenols

The protocol involved an oral, a gastric, and an intestinal stage as reported by Minekus et al. [14]. The cooked-to-optimum pasta samples (i.e., 5.0 g) were sequentially hydrolyzed at 37 °C through (i) an oral phase, (5 mL of salivary fluid at pH = 7.0 plus human salivary α-amylase (A1031; Sigma-Aldrich; Milan, Italy; 75 U/mL) for 2 min; (ii) a gastric phase (10 mL of a simulated gastric fluid at pH 3.0 plus pepsin (P7012; Sigma-Aldrich; 2.000 U/mL) for 120 min; and (iii) an intestinal phase (20 mL of simulated intestinal fluid at pH = 7.0 plus pancreatin (P7545; Sigma-Aldrich; Milan, Italy; 100 U/mL) and bile salts (B8631; Sigma-Aldrich; Milan, Italy; 10 mM) for a further 120 min. Appropriate amounts of HCl (1 M) and NaOH (1 M) were added for the pH adjustment. Liquid aliquots were carefully removed from each hydrolyzed sample after each hydrolysis phase and stored at −20 °C.

#### 2.3.2. Nutritional Starch Fractions Determination

The rapidly digestible starch (RDS) and slowly digestible starch (SDS) were measured with the method of Englyst et al. [15], with minor modifications as detailed by Simonato et al. [5]. The RDS and SDS contents were calculated considering the glucose released after 20 min and 120 min of incubation [15] by measuring the amount of glucose spectrophotometrically using a D-Glucose assay kit (GOPOD, Megazyme, Wicklow, Ireland). The resistant starch (RS) was quantified by a K-RSTAR assay kit (Megazyme, Wicklow, Ireland). The total starch content was calculated as the sum of non-resistant starch and RS following the K-RSTAR assay kit’s instructions.

#### 2.3.3. Starch Hydrolysis Index

The cooked-to-optimum spaghetti samples (100 mg) were dispersed in 4 mL of maleic buffer (pH 6), containing an enzyme mixture composed of amyloglucosidase (AMG; 4 μL; 300 U/mL; Megazyme, Wicklow, Ireland) and pancreatic α-amylase (40 mg; 3000 U/mg; Megazyme, Wicklow, Ireland). Samples were incubated in a shaking water bath at 37 °C. At selected time intervals (i.e., 0, 30, 60, 120, and 180 min) the reaction was stopped by adding absolute ethanol. Samples were then centrifuged at 2500× *g* for 10 min. The amount of glucose was quantified as previously detailed, after the correction for glucose present in the AMG solution. Values were plotted on a graph vs. time, and the area under the hydrolysis curve (AUHC; 0–180 min) was measured by using the trapezoid rule. A starch hydrolysis index (HI) value was calculated as the AUHC with the product as a percentage of the corresponding area with white wheat bread [16].

### 2.4. Extraction and Characterization of Untargeted Phenolic Profile by UHPLC-ESI/QTOF Mass Spectrometry

Three replicates (1.0 g) for each cooked-to-optimum pasta batch were extracted in 10 mL of a methanol/water 80:20 (*v/v*) solution, by using a homogenizer-assisted extraction with an Ultra-Turrax (Ika T25, Staufen, Germany; 5000× *g*; 3 min) [7]. The extracts were centrifuged (10,000× *g*; 10 min; 4 °C), filtered (0.22 μm cellulose syringe filters), and collected [7]. The bound phenolic fraction was extracted from the remaining solid residue [17]. After the alkaline hydrolysis (3 mL of 2 M sodium hydroxide; 1 h; room temperature), the pH was adjusted to 3 with 3 M citric acid and the bound phenolics were extracted with 8 mL of ethyl acetate. After 15 min at 6500 rpm centrifugation, 4 mL of the supernatant was dried under a nitrogen flow at 55 °C and the residue was dissolved in 1 mL of 1% formic acid in 80% methanol, vortexed, and centrifuged (10,000× *g* for 10 min). The resulting solution was filtered (0.22 μm cellulose syringe filters) and 200 μL aliquot was transferred to amber vials for analysis.

The modifications in the polyphenol profile after subjecting the cooked samples through the in vitro static digestion method (i.e., Section 2.3.1) were evaluated by ultra-high-performance liquid chromatography-quadrupole time-of-flight (UHPLC-ESI/QTOF) mass spectrometry [7]. Liquid aliquots collected after the oral, the gastric, and the pancreatic in vitro digestion phases were centrifuged at 7000× *g* for 10 min and then filtered (0.22 μm cellulose syringe filters). A mixture of water and acetonitrile (VWR, Milan, Italy; both acidified with 0.1% formic acid) as a mobile phase and an Agilent Zorbax Eclipse-plus C18 column (100 mm × 2.1 mm, 1.8 μm) were used. The gradient was from 6% acetonitrile to 94% acetonitrile in 30 min and the flow rate was 0.220 mL/min. The mass spectrometer worked in the positive scan mode (100–1200 *m/z*), injecting 6 μL and source conditions were: sheath gas nitrogen 10 L min^−1^ at 350 °C; drying gas 10 L min^−1^ at 280 °C; nebulizer pressure 60 psig, nozzle voltage 300 V, capillary voltage 3.5 kV. Three technical replicates were done for each pasta batch

The software Agilent Profinder B.06 was used to elaborate the raw features. Features were aligned, and the monoisotopic accurate mass was combined with the isotopic profile for the compounds’ annotation, thus reaching a level 2 of confidence in annotation (i.e., putatively annotated compounds) [18]. The database Phenol-Explorer 3.6 was used. The mass accuracy tolerance was set to 5 ppm. Phenolic compounds passing the frequency of the detection thresholds (100% of replications within at least one condition) were classified and then quantitative information was produced using calibration curves (in the range 0.05–500 mg/L) from standard solutions of the single phenolic compounds (purchased from Extrasynthese; Genay; France, purity >98%). Selected representative compounds were as follows: cyanidin, quercetin, luteolin, catechin, tyrosol, and ferulic acid. Results were expressed as mg phenolic equivalents/g dry matter (DM). The polyphenols’ bioaccessibility was calculated [19]:

Bioaccessibility = (PCA/PCB) × 100

where PCA is the total phenolic subclass content in the samples (mg/g DM) collected after each individual in vitro digestion incubation phase, and PCB is the total phenolic subclass (free plus bound polyphenols) content in the cooked samples before the in vitro digestion.

### 2.5. Statistical Analysis

Analyses were run in triplicate on each batch and data were expressed as mean values ± standard deviation. The data were evaluated using a one-way analysis of variance (ANOVA). Differences among means were evaluated by Tukey’s HSD tests (*p* < 0.05). The statistical software was R project (version 3.2.3, December 2015). Metabolomic data were pre-processed using the software Agilent Mass Profiler Professional B.12.06 (Agilent Technologies, Santa Clara, CA, USA). Compounds were aligned and filtered by abundance (peak area >5000 counts), normalized at the 75th percentile, and baselined against the median [7]. The metabolomics-based dataset was then exported into SIMCA 13 (Umetrics, Malmo, Sweden) to produce a supervised orthogonal projection to latent structures discriminant analysis (OPLS-DA) model. Hotelling’s T2 together with CV-ANOVA (*p* < 0.01) and permutation testing were checked to cross-validate the model. Model parameters (i.e., R^2^Y and Q^2^Y) were recorded. The variable selection method variable importance in projection (VIP) was used to point out the phenolic compounds with the highest discrimination ability (VIP score >1) during the in vitro digestion.

## 3. Results and Discussion

### 3.1. Moisture Content, Water Activity and Optimal Cooking Time of Samples

The moisture content and the a_w_ values of the fresh pasta samples were on average 30.8 g water/100 g of fresh pasta and 0.96, respectively, and were not influenced by the inclusion of MOLP (*p* > 0.05; data not reported). The gradual substitution of semolina flour with MOLP induced a progressive reduction in the OCT, ranging from 5 min for the M0 to 2.5 min for the M15 pasta samples (i.e., 4 min and 3.5 min for M5 and M10, respectively). The progressive decrease in the OCT as a function of the MOLP inclusion level could be due to the great presence of fiber (30.7 g/100 g product) in the MOLP along with the reduction in the total starch content of the samples. For instance, fiber inclusion in wheat pasta can affect the starch–gluten structure, allowing a faster cooking water entrance in the core of the pasta and a resultant faster starch granule gelatinization, thus reducing the OCT [5,20].

### 3.2. Free and Bound Phenolic Profiles of Cooked-To-Optimum Samples

On the cooked pasta samples, 152 phenolic compounds were putatively annotated, being 38 flavone equivalents (mainly flavones and flavanones), 30 flavonols, 4 flavan-3-ols, 27 anthocyanins, 36 phenolic acids, and 17 remaining compounds. A comprehensive list of each phenolic compound annotated is provided in File S1, considering both the mass abundances and composite spectra. The most abundant compounds detected were tetramethylscutellarein (a flavone), glycosidic and isomeric forms of quercetin and kaempferol (belonging to flavonols), pyrogallol (a low molecular weight phenolic, characterizing mainly the bound phenolic fraction), and malvidin 3-*O*-galactoside (an anthocyanin).

**Figure 1:**
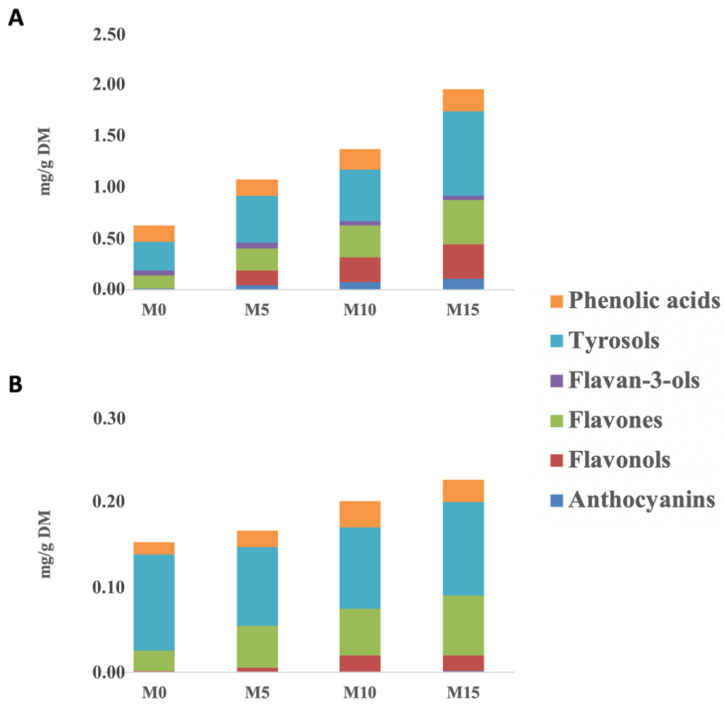
Cumulative phenolic composition (as mg phenolic equivalents/g dry matter) in the cooked pasta samples, considering the free (**A**) and bound (**B**) phenolic fractions. M0: 100% durum wheat semolina fresh pasta. M5, M10, and M15 = Fresh pasta produced with 5, 10, and 15 g/100 g *w/w Moringa oleifera* L. leaf powder, respectively.

Thereafter, the main phenolic classes characterizing the different cooked samples were targeted. The results are reported in Figure 1, considering both the free (A) and the bound (B) phenolic contents.

Overall, the greatest (*p* < 0.05) total phenolic content (i.e., sum of the different phenolic classes) was found in the M15 sample, being 2.19 mg/g DM, followed by the M10 (1.58 mg/g DM), M5 (1.24 mg/g) and M0 pasta samples (0.78 mg/g DM), as a function of the increasing inclusion level of MOLP in the formulation. Interestingly, the highest inclusion level of MOLP (i.e., 15% *w/w*) was also found to impact the bound phenolic content of the cooked pasta samples (Figure 1B). When considering the specific phenolic composition of the different cooked samples, it was evident that the most represented phenolic classes (in terms of semi-quantitative contents) were low-molecular weight phenolic compounds grouped as tyrosol equivalents (according to the Phenol-Explorer Database), followed by flavonoids (mainly flavonol and flavone equivalents) and phenolic acids. In addition, the highest increase of polyphenols was observed in the M15 sample when considering the total flavonol equivalents; in fact, this class of compounds moved from 0.19 mg/100 g DM for M0 to 35.73 mg/100 g DM for the M15 sample.

Another phenolic class affecting the phenolic profile of the different cooked pasta samples was tyrosol equivalents. These low-molecular weight phenolics were greater in MOLP-substituted spaghetti when compared to M0. These differences may be related to the inherent phenolic profile of MOLP, along with the specific inclusion level in each recipe. Moringa leaves have been reported as a great source of polyphenols, such as flavonoids [7]. Likewise, an abundance of glycosidic forms of quercetin and kaempferol equivalents (i.e., flavonols), followed by hydroxycinnamic/hydroxybenzoic acids and low-molecular-weight phenolics (i.e., phlorin and protocatechuic aldehyde) has been previously indicated [7]. In addition, previous studies reported that cooking by boiling can cause substantial water-losses and/or oxidative degradation of several antioxidant components [21]. According to the literature, whole cereal grains (such as wheat) are reported to be abundant in bound phenolic compounds, such as phenolic acids (i.e., ferulic acid) and lignans. In fact, these compounds are particularly concentrated in the external bran tissues [22]. However, in this work, we found that the M0-cooked sample was characterized by a lower phenolic content, also when considering the bound phenolic composition (Figure 1). Overall, the trends observed for the M0 sample could be explained by considering the different variables such as (a) the milling process conditions, widely reported as one of the major factors able to affect the phenolic profile of durum wheat semolina [23]; (b) the cooking-by-boiling process used; and (c) the rupture of the plant cell structures as promoted by the extraction method, based on a homogenizer-assisted extraction [7].

### 3.3. Changes of Phenolic Profiles during In Vitro Static Digestion

The cooked-to-optimum samples were hydrolyzed through a standardized static digestion method, with the aim to describe the changes in the phenolic profile. In particular, 102 phenolic compounds were found. Flavonoids were the most represented (56 compounds), followed by hydroxycinnamic acids (24 compounds) and tyrosol equivalents. Overall, the phenolic compounds exhibited different bioaccessibility behaviors, mainly imposed by the presence of different food components (e.g., dietary fiber) in each pasta sample, in line with previous findings [8,10,21]. As can be observed in Table 1, lower percentage bioaccessibility values were detected during the entire gastrointestinal process for specific subclasses of compounds, namely anthocyanins, flavanols, and flavonols.

On the other hand, flavones, tyrosols, and phenolic acids had a moderate bioaccessibility during the in vitro digestion process (mainly after 120 min of the pancreatic step). In fact, the higher percentage bioaccessibility values were measured for the tyrosol equivalents in the M0 sample (i.e., 29.21%), followed by flavones characterizing the M5 sample (i.e., 28.26%). However, a linear trend between the polyphenols’ bioaccessibility and MOLP increasing levels in the recipe was not observed. The similarities in percentage of bioaccessibility are expected because the polyphenol content was increased but the in vitro digestive conditions were the same.

Recent studies showed that several bioactivities including antioxidant, antiproliferative, immuneregulatory, hormonal regulation abilities, and neuro-/hepato-/cardioprotective effects can be related to the consumption of phenolic-rich foods [21]. However, these health benefits are greatly dependent on the bioaccessibility potential within the human digestive tract. Present findings corroborated the fact that food components–polyphenols interactions should be considered when studying the changes in the bioaccessibility values during the in vitro digestion in a real food system (i.e., cooked matrix) [8]. Another important factor is the impact of the cooking process, that can modify the bioaccessibility of several phenolic compounds [24,25]. Therefore, the relatively high-percentage bioaccessibility values observed in the pancreatic phase for some phenolic classes (such as phenolic acids, flavones, and tyrosol equivalents) are not surprising and could promote an antioxidant environment in the digestive tract [26]. According to the literature [21,27], the detected bioaccessibility trends may be related to the simulated gastrointestinal digestion conditions used. These latter are not only responsible to break down food matrices and thus release bound phenolic compounds but may also modify the phenolic hydroxyl group (major functional group) of the released phenolics, thus leading to a decrease or increase in the phenolic content in the digestion fluids. In addition, according to the phenolic profile reported for wheat flour, we found a greater bioaccessibility of alkylphenols (quantified as tyrosol equivalents) for the M0 sample during the intestinal step (File S1). In particular, greater percentage bioaccessibility values were measured for three wheat compounds, namely 5-heneicosenylresorcinol, 5-heneicosylresorcinol, and 5-tricosenylresorcinol, which are widely reported as the most abundant in wheat flour [28]. Present findings are difficult to compare with the literature, due to the lack of similar works. To the best of our knowledge, only the work by Caicedo-Lopez and co-authors [12] investigated the changes in the bioaccessibility, intestinal permeability, and antioxidant capacity of the free-phenolic fraction of MOLP after an in vitro gastrointestinal digestion. The authors showed that the greatest content of bioactive compounds was retained in the non-digestible fraction, with higher bioaccessibility values recorded for some phenolics acids, morin, and kaempferol, in line with our findings.

**Figure 2:**
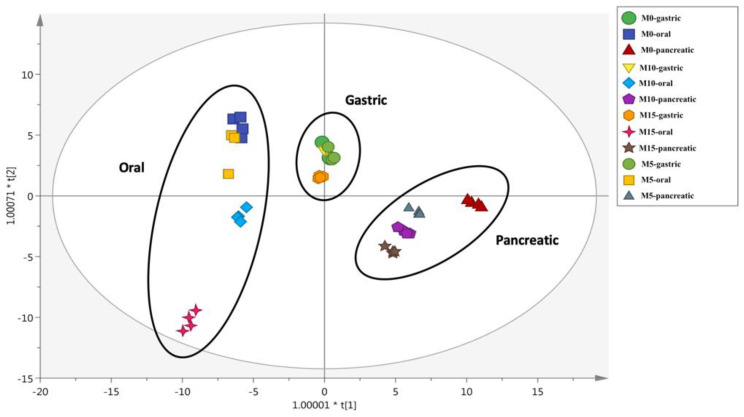
Orthogonal projections to latent structures discriminant analysis (OPLS-DA) score plot on the cooked pasta samples’ phenolic profile after oral, gastric, and pancreatic phases of in vitro static digestion. M0: wheat semolina fresh pasta. M5, M10, and M15: fresh pasta produced with 5, 10, and 15 g/100 g *w/w* MOLP, respectively.

Multivariate statistics based on OPLS-DA-supervised modelling was then applied to the metabolomics-based dataset. The OPLS-DA score plot built considering each hydrolyzed cooked sample is reported in Figure 2.

Most of the sample variability characterized the oral and the pancreatic phases of the in vitro static digestion, whilst the gastric phase clustered together. The oral-digested M15 sample clustered differently when compared with the others, likely due to the greater content of anthocyanins and flavonol equivalents (i.e., 0.02 and 0.06 mg/100 g DM, respectively). Interestingly, the OPLS-DA score plot confirmed a characteristic phenolic profile for the M0 spaghetti after the pancreatic phase, likely due to its inherent phytochemical composition when compared with the MOLP-substituted counterparts. The OPLS-DA score plot was also inspected for model accuracy parameters recording the acceptable goodness of fit/prediction values (i.e., R^2^X = 0.92; R^2^Y = 0.66; Q^2^cum = 0.50). Finally, the VIP method was used to rank those phenolic compounds most affected during the in vitro digestion. A list containing these VIP markers is reported in File S1, together with the corresponding VIP score (i.e., their discrimination potential) and standard error. Overall, 41 compounds were detected, including flavonoids (such as glycosidic forms of flavonols and flavones, followed by anthocyanins) and phenolic acids (i.e., hydroxycinnamic acids). The highest VIP scores (i.e., representing those compounds most affected by the in vitro static digestion process) were recorded for stigmastanol ferulate (1.23), isomeric and glycosidic forms of kaempferol (1.19), and other flavones (such as luteolin 7-*O*-rutinoside, apigenin 6,8-di-*C*-glucoside, and chrysoeriol 7-*O*-apiosyl-glucoside). Interestingly, the VIP selection method revealed the presence of several anthocyanins, including acetyl-glycosidic forms of peonidin, malvidin, and petunidin. Regarding the other VIP markers discriminating the in vitro digestion process, we found a good distribution of flavones. In this regard, luteolin (VIP score = 1.16) was clearly related to the presence of MOLP in the recipe, being only detected in the M5, M10, and M15 samples (File S1). Similar findings were obtained for quercetin (presenting a VIP score = 1.08), which was found to be abundant in the MOLP-substituted samples during the pancreatic phase.

### 3.4. In Vitro Starch Digestion of Cooked Samples

The nutritional starch fraction contents, along with the HI of the cooked samples, are reported in Table 2.

An increase in the SDS and a decrease in the RDS fractions were reported considering the gradual substitution of durum wheat semolina with MOLP. The M10 and M15 cooked pasta samples exhibited the lowest RDS value (i.e., 38.1 and 34.1 g/100 g DM; *p* < 0.05) along with the greatest SDS content (i.e., 18.1 and 20.8 g/100 g DM; *p* < 0.05), when compared with the other samples. From a nutritional standpoint, the RDS fraction was found to be responsible for a rapid increment in the blood glucose levels in humans, while the SDS fraction, being characterized by slow digestion properties, can provide a prolonged release of glucose over time [29]. The mechanism by which the MOLP addition affected the nutritional starch fraction contents of the samples may depend on the interactions among the major constituents (i.e., protein, starch, and fibre polysaccharides) and other minor compounds (i.e., certain classes of dietary polyphenols) [5,8,11,20]. It has been reported that the inclusion of fiber-rich ingredients could modulate the in vitro starch digestion to a certain extent, by changing both the physicochemical properties of the food system [5,30]. For instance, a reduction in the RDS fraction, along with an increment in the SDS fraction exerted by the addition of olive pomace has been reported in wheat-based spaghetti [5]. Lastly, the RS represents, by definition, a certain fraction of starch not degraded in the human small intestine but fermented in the large intestine, with a series of physiological benefits [31]. The RS content of the M0 pasta (i.e., 2.1 g/100 g DM) appeared in line with previous findings for similar food products [32]. However, the RS content of the samples decreased with the increasing inclusion level of MOLP in the recipe, with the lowest value recorded for M15 (i.e., 1.1 g/100 g DM; *p* < 0.05) (Table 2). A possible explanation is that the added MOLP could have contributed to undermine the compact microstructure of wheat pasta, by allowing water and heat to more easily penetrate the pasta during cooking, thus contributing to gelatinize the resistant starch granules present in the core region of the pasta strand to a greater extent [20,33]. In addition, the RS fraction in durum wheat pasta is mainly formed during the pasta extrusion at a high temperature and during the drying process, which in our case was not made [34]. Lastly, the gradual substitution of wheat semolina with MOLP decreased the total starch content of the samples (*p* < 0.05), probably due to a dilution effect of starch exerted by the addition of MOLP, as a consequence of the individual chemical compositions of the selected ingredients.

The starch HI can be used as a predictor of the in vivo glycemic response for a certain starch-based food product [35]. In addition, from the HI values, it is possible to calculate the glycemic index of starch-based foods by applying predictive equations [16]. As reported in Table 2, using white wheat bread as a reference, the HI of the M0 pasta was 47.4. The substitution of a part of the durum wheat semolina with increasing levels of MOLP decreased (*p* < 0.05) the HI of the cooked pasta, the lowest values being recorded for M15 (i.e., 41.8; *p* < 0.05). The decrease in the HI values as a function of the substitution level of MOLP in the recipe could be related to the decrease in the starch content for the replacement of semolina with different quantities of MOLP, as also described in the literature [5], or to the interplay of several factors related to the inherent chemical compositions of the samples. In particular, MOLP contains greater amounts of dietary fiber (about 30.7 g/100 g) and protein (about 29.9 g/100 g) than durum wheat semolina. This may have contributed to entrap starch granules into a non-starchy network with a limited enzyme accessibility [35,36]. Accordingly, cookies enriched with increasing amounts of alfalfa seed (*Medicago sativa* L.) flour showed a reduction in the in vitro starch digestibility compared with the control [37]. Furthermore, the specific phenolic composition characterizing the MOLP-substituted cooked pasta samples (Figure 1; File S1) could have contributed to modulate, at least in part, the in vitro starch digestion of the samples. In particular, certain classes of phenolic compounds may have a role in modulating the in vitro starch digestion, via the inhibition of the starch digestive enzymes (i.e., α-amylase and α-glucosidase enzymes) and/or through the non-covalent interactions with starch on cooking, thus contributing to the formation of inclusion and non-inclusion starch-complexes with a limited enzyme accessibility [11,38,39]. For instance, secondary metabolites characterizing MOLP-substituted pasta (e.g., flavones, flavonols, and hydroxycinnamic acids; File S1) have already been reported to inhibit both α-glucosidase and α-amylase during in vitro activities [40,41,42].

## 4. Conclusions

Fresh pasta was formulated by replacing durum wheat semolina with 0, 5, 10, and 15 g/100 g *w/w* of MOLP. After cooking and following an in vitro digestion process, the phenolic compounds exhibited different bioaccessibility behaviors, with an increase in bioaccessibility observed for flavonols characterizing the digested pasta sample formulated with the greatest inclusion level of MOLP in the recipe. Multivariate statistics highlighted a general abundance of flavonoids and phenolic acids among the discriminant markers. Additionally, the inclusion of MOLP in the pasta influenced the rate of in vitro starch digestion in the cooked samples, showing an increase in the SDS fraction, and a decrease in the RDS fraction and HI values. Taken together, the present findings support the fact that MOLP may represent a valuable ingredient to produce a functional pasta. Future investigations considering technological and sensorial aspects are needed to expand the knowledge on the effect of an MOLP addition in fresh pasta formulation.

## Figures and Tables

**Table 1:** Bioaccessibility values (expressed as % of phenolic equivalents) for the different phenolic subclasses during the in vitro static digestion of the cooked-to-optimum pasta samples formulated with different substitution levels of *Moringa **o**leifera* L. leaf powder (MOLP), considering the oral, gastric, and pancreatic phases.

Phenolic Subclasses	Pasta Samples	TPC Cooked Samples (mg Eq./100 g)	% Bioaccessibility		
			Oral	Gastric	Pancreatic
Anthocyanins	M0	0.87 ± 0.04 ^a^	0.31	0.45	nd
	M5	4.47 ± 0.22 ^b^	0.14	0.13	0.07
	M10	7.43 ± 0.36 ^c^	0.17	0.19	0.37
	M15	10.41 ± 0.50 ^d^	0.22	0.20	0.27
Flavonols	M0	0.19 ± 0.01 ^a^	nd	nd	nd
	M5	14.74 ± 0.72 ^b^	0.04	0.22	1.01
	M10	26.06 ± 1.20 ^c^	0.09	0.26	1.13
	M15	35.73 ± 1.80 ^d^	0.16	0.44	1.14
Flavones	M0	15.19 ± 0.71 ^a^	13.08	9.31	5.53
	M5	26.59 ± 1.33 ^b^	7.48	4.69	28.26
	M10	36.70 ± 1.82 ^c^	5.01	3.10	17.27
	M15	50.28 ± 2.41 ^d^	3.23	2.13	15.16
Flavan-3-ols	M0	4.36 ± 0.18	14.45	nd	nd
	M5	5.20 ± 0.21	7.05	nd	nd
	M10	3.91 ± 0.12	5.52	nd	nd
	M15	4.01 ± 0.13	8.50	nd	nd
Tyrosols	M0	39.25 ± 1.90 ^a^	1.92	1.89	29.21
	M5	55.11 ± 2.71 ^b^	1.41	1.93	13.80
	M10	60.11 ± 2.98 ^b^	1.30	1.73	8.32
	M15	93.53 ± 4.65 ^c^	0.87	1.17	3.01
Phenolic acids	M0	17.96 ± 0.86 ^a^	3.30	3.10	12.45
	M5	17.95 ± 0.89 ^a^	2.42	4.10	12.36
	M10	23.50 ± 1.18 ^b^	1.81	3.09	8.97
	M15	24.86 ± 1.25 ^b^	1.86	3.22	8.28

M0: wheat semolina fresh pasta. M5, M10, and M15: fresh pasta produced with 5, 10, and 15 g/100 g *w/w* MOLP, respectively. nd = not detected. Within each subclass, means within a column with different superscript letters for the total phenolic content (TPC) of the cooked samples differed at p < 0.05.

**Table 2:** Starch fractions (g/100 g dry matter), total starch (g/100 g dry matter), and in vitro hydrolysis index (HI) of the cooked-to-optimum pasta samples formulated with different substitution levels of *Moringa oleifera* L. leaf powder (MOLP). Results are expressed as mean ± standard deviation (*n* = 3).

	Substitution with MOLP			
	M0	M5	M10	M15
Rapidly digestible starch	44.3 ± 0.31 ^a^	43.8 ± 0.79 ^a^	38.1 ± 1.76 ^b^	34.1 ± 3.49 ^b^
Slowly digestible starch	16.8 ± 0.70 ^a^	16.8 ± 0.67 ^a^	18.1 ± 0.20 ^b^	20.8 ± 0.67 ^c^
Resistant starch	2.1 ± 0.26 ^a^	1.4 ± 0.04 ^b^	1.3 ± 0.01 ^c^	1.1 ± 0.04 ^d^
Total starch	63.1 ± 1.33 ^b^	62.1 ± 1.77 ^b^	57.7 ± 1.21 ^a^	55.9 ± 1.44 ^a^
HI ^1^	47.4 ± 1.05 ^a^	45.4 ± 1.32 ^ab^	43.9 ± 1.21 ^ab^	41.8 ± 0.81 ^b^

Values in the same row with different superscripts are significantly different (*p* < 0.05). M0: wheat semolina fresh pasta. M5, M10, and M15: fresh pasta produced with 5, 10, and 15 g/100 g *w/w* MOLP, respectively. ^1^ Calculated using commercial white wheat bread as a reference.

## Supplementary Material

The following are available online at https://www.mdpi.com/2304-8158/9/5/628/s1, File S1: Metabolomics dataset containing polyphenols identified by UHPLC-QTOF mass spectrometry, together with semi-quantitative values for each phenolic class and VIP markers following OPLS-DA multivariate model.
